# Supplementary material for: Dynalign II: common secondary structure prediction for RNA homologs with domain insertions
Source: Nucleic Acids Res. 2014 Nov 21;42(22):13939–48. doi: 10.1093/nar/gku1172 (PMC4267632; doi:10.1093/nar/gku1172)
Supplement: SUPPLEMENTARY DATA [file supp_gku1172_nar-02021-z-2014-File012.zip › manual/Text/PARTS.html]

RNAstructure Command Line Help: PARTS


|  |  |  |
| --- | --- | --- |
|  | RNAstructure Command Line Help PARTS | - Contents - Index |
| PARTS takes two homologous sequences as input and aligns the two sequences and predits base pairing probabilities for conserved structures. It can run in three modes: map (maximum a posteriori) mode to find a maximum probability structure, pp (pair probability) mode, which yields a matrix of base pairing probabilities, or stochsample (stochastic sampling) to sample generated structures. USAGE: PARTS <configuration file> Required parameters: <configuration file> \| The name of a file containing required configuration data. Options which don't require added values: NONE Options which require added values: NONE Configuration file format: The following is a description of valid options allowed in the configuration file. ``` ################################################################ # IMPORTANT CONFIG FILE FORMAT NOTES: # # Config file options described below are case sensitive. # # Option lines may be specified by the option name followed by a space and the option's desired value. # When specifying an option, there may be nothing else on the line. # <option> <value> # # Specifying comment lines: # Comment lines must begin with "#" followed by a space. # There may not be more than one "#" in a comment line. # However, a comment line may be an unbroken string of "#", as in a divider between sets of options. # # Blank lines are skipped. ################################################################ ################################################################ # Required input ################################################################ seq1 seq1.seq seq2 seq2.seq mode map (or pp or stochsample) ################################################################ # Output files; if these aren't specified defaults are used. ################################################################ # Output file for sequence 1 in pp mode seq1_pp_op ../my_pp1 # Output file for sequence 2 in pp mode seq2_pp_op my_pp2 # Output ct for sequence 1 in stochsample mode seq1_sample_ct_op ../my_ct1_sample.txt # Output ct for sequence 2 in stochsample mode seq2_sample_ct_op ../../my_ct2_sample.txt # Output alignment file for stochsample mode sample_aln_op ./my_aln_sample.txt # Output ct for sequence 1 in map mode seq1_map_ct_op ../my_map_ct1.txt # Output ct for sequence 2 in map mode seq2_map_ct_op ../../my_map_ct2.txt # Output alignment file for map mode map_aln_op ../my_map_aln.txt ################################################################ # Other parameters ################################################################ # Number of structures to sample in stochsample mode nsamp 1000 						 ``` References: 1. Reuter, J.S. and Mathews, D.H. "RNAstructure: software for RNA secondary structure prediction and analysis." *BMC Bioinformatics*, 11:129. (2010). 2. Harmanci, A.O., Sharma, G. and Mathews, D.H. "Stochastic sampling of the RNA structural alignment space." *Nucleic Acids Res.*, 37:4063-4075. (2009). 3. Harmanci, A.O., Sharma, G. and Mathews, D.H. "PARTS: Probabilistic Alignment for RNA joinT Secondary structure prediction." *Nucleic Acids Res.*, 36:2406-2417. (2008). | | |
| Visit The Mathews Lab RNAstructure Page for updates and latest information. | | |
